# Study of Binding Interaction between Pif80 Protein Fragment and Aragonite

**DOI:** 10.1038/srep30883

**Published:** 2016-08-03

**Authors:** Yuan-Peng Du, Hsun-Hui Chang, Sheng-Yu Yang, Shing-Jong Huang, Yu-Ju Tsai, Joseph Jen-Tse Huang, Jerry Chun Chung Chan

**Affiliations:** 1 Department of Chemistry, National Taiwan University, No. 1, Section 4, Roosevelt Road, Taipei 106, Taiwan; 2 Instrumentation Center, National Taiwan University, No. 1, Section 4, Roosevelt Road, Taipei 106, Taiwan; 3 Institute of Chemistry, Academia Sinica, No. 128, Sec. 2, Academia Road, Nankang, Taipei 115, Taiwan

## Abstract

Pif is a crucial protein for the formation of the nacreous layer in *Pinctada fucata*. Three non-acidic peptide fragments of the aragonite-binding domain (Pif80) are selected, which contain multiple copies of the repeat sequence DDRK, to study the interaction between non-acidic peptides and aragonite. The polypeptides DDRKDDRKGGK (Pif80-11) and DDRKDDRKGGKDDRKDDRKGGK (Pif80-22) have similar binding affinity to aragonite. Solid-state NMR data indicate that the backbones of Pif80-11 and Pif80-22 peptides bound on aragonite adopt a random-coil conformation. Pif80-11 is a lot more effective than Pif80-22 in promoting the nucleation of aragonite on the substrate of β-chitin. Our results suggest that the structural arrangement at a protein-mineral interface depends on the surface structure of the mineral substrate and the protein sequence. The side chains of the basic residues, which function as anchors to the aragonite surface, have uniform structures. The role of basic residues as anchors in protein-mineral interaction may play an important role in biomineralization.

Biominerals are composite materials comprising inorganic minerals and biomolecules^1^. More than sixty different types of biominerals have been identified in 55 phyla of living organisms^2^,^3^,^4^. These biominerals typically demonstrate higher mechanical strengths and more intriguing architectures than their geological counterparts^5^. Aragonite and calcite, which are the two polymorphs of CaCO_3_, constitute the nacreous and prismatic layers of mollusks shells, respectively^6^. Although it has been established that macromolecules such as proteins are crucial for the crystallization of CaCO_3_ *in vivo*^7^,^8^,^9^, how nature precisely controls the formation of the mineral phases of different thermodynamic stabilities in mollusks is not well understood^10^,^11^,^12^. AP7, AP24, N16, and Pif are matrix proteins identified in the nacreous microstructure of several mollusks^6^. Polypeptides derived from AP7, AP24, and N16 have been extensively studied with respect to their relevance to the crystallization process of CaCO_3_^13^,^14^,^15^,^16^,^17^,^18^,^19^,^20^,^21^. In particular, it has been shown that the synergy of proteins and the substrate of β-chitin is important for the nucleation of aragonite^8^,^17^. The matrix protein Pif, which consists of a chitin-binding (Pif97) and an aragonite-binding (Pif80) domains, plays a key role in the formation of nacre in *Pinctada fucata*^22^. However, relatively few *in vitro* studies of Pif have been reported to date^22^,^23^. While the Pif97 domain is highly conserved in the homologs identified in different species, the sequence of the Pif80 domain has a remarkable variation^6^. It would be of great interest to study the model peptides derived from Pif80 to unravel the underlying physical principles governing the binding interaction between Pif80 and aragonite.

Among the 460 residues of the Pif80 domain, there are 17 repeats of the motif of Asp-Asp-Arg(Lys)-Lys(Arg), scattering throughout the entire sequence. As our first model peptide for the study of Pif80, we selected the sequence of Pif80_913−934_ (Pif80-22), which is the only region in Pif80 containing two tandem repeats of the DDRK motif. In addition, we prepared two shorter peptides, viz. Pif80-7 and Pif80-11, to study whether the binding affinity to aragonite would depend on the sequence length. Our experimental data indicated that Pif80-22 and Pif80-11 had higher binding affinity to aragonite than Pif80-7 and that Pif80-11 had the highest propensity to induce the crystallization of aragonite on a β-chitin substrate. Solid-state NMR measurements revealed that the backbone conformation of Pif80-11 and Pif80-22 remained to be random-coiled upon their binding to aragonite and that the basic residues played a prominent role in their interaction with aragonite.

## Results

### Effect of Pif80-x on the Crystal Morphology of Calcium Carbonate

**Figure 1:**
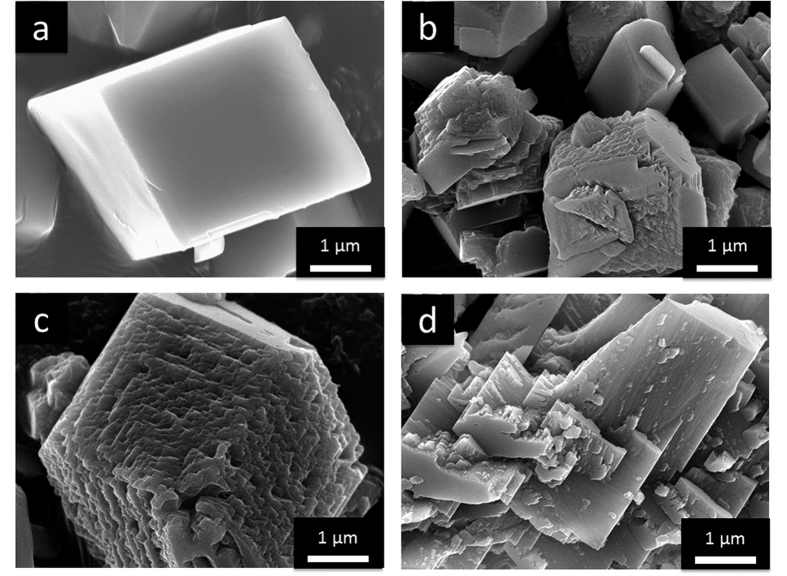
SEM images of calcite crystallites prepared in the gas diffusion experiments, where the solution of 100 mM of CaCl_2_ contained: (**a**) no peptide, (**b**) Pif80-7, (**c**) Pif80-11, and (**d**) Pif80-22.

Three peptide fragments of Pif80 with the sequences of DDRKGGK (Pif80-7), DDRKDDRKGGK (Pif80-11), and DDRKDDRKGGKDDRKDDRKGGK (Pif80-22) were prepared by solid-phase synthesis and then characterized by mass spectrometry (Supporting Information, Figs S1 and S2). As shown in the circular dichroism (CD) spectra, all of the Pif80-*x* peptides adopted a random-coil conformation in the aqueous solutions of CaCl_2_ and MgCl_2_ (Supporting Information, Fig. S3). For the crystallization assay, a solution of CaCl_2_ (100 mM) and Pif80-*x* (0.1 mM) was stirred under the atmosphere of NH_3(g)_ and CO_2(g)_ for 24 h. The X-ray diffraction (XRD) analysis indicated that the collected precipitates were calcite (Supporting Information, Fig. S4). In the subsequent discussion, the samples are referred to as Pif80-*x*/calcite. The isoelectric points (pI) of Pif80-7, Pif80-11, and Pif80-22 were estimated to be 10.6, 10.3, and 10.3, respectively^24^. Thus, Pif80-*x* were all positively charged in the solution because the final pH of the solution was 9.2. The scanning electron microscopic (SEM) images revealed that the Pif80-*x* peptides affected the morphology of the crystallites of calcite to various degrees (Fig. 1). In the cases of Pif80-7/calcite and Pif80-11/calcite, although the crystal surfaces were modified considerably, the characteristic rhombohedral shapes of calcite were largely retained. For Pif80-22/calcite, on the other hand, the morphology of the crystallites became rather irregular. The greater effect of Pif80-22 was not unexpected because this longer peptide contained more charged amino acids than Pif80-7 and Pif80-11.

**Figure 2:**
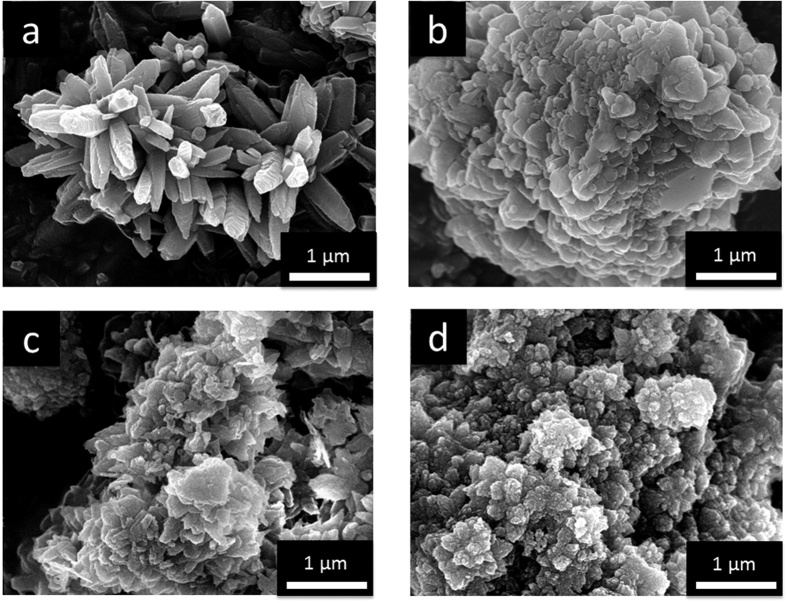
SEM images of aragonite crystallites prepared in the gas diffusion experiments, where the solution of 100 mM of CaCl_2_ and 100 mM of MgCl_2_ contained: (**a**) no peptide, (**b**) Pif80-7, (**c**) Pif80-11, and (**d**) Pif80-22.

To facilitate the crystallization of aragonite, we repeated the gas diffusion experiment in the presence of Mg^2+^ ions^25^,^26^. XRD results confirmed that the samples obtained were aragonite (Supporting Information, Fig. S5), which are henceforth referred to as Pif80-*x*/aragonite. Figure 2 shows the SEM images of the aragonite crystallites. For the control sample, which was prepared in the absence of peptides, the crystal shape was spine like. Each “spicule” was formed by the stacking of tiny plate-like crystallites, which suggests that there is a structural ordering regarding the relative orientation of the tiny crystallites. By contrast, the crystallites obtained in the presence of Pif80-*x* were less ordered in shape and size. It is possible that the interactions between Pif80-*x* and aragonite were so strong that the peptides served as effective binders between crystallites. Thus, the crystal growth became largely kinetically controlled, which led to a disordered aggregation of the crystal grains. In addition, the Pif80-*x* peptides bound on the crystallite surface would inhibit the crystal growth. Thus, the weaker interaction between Pif80-7 and aragonite is evident from the larger grain size (Supporting Information, Fig. S5). Attempts to determine the surface area per unit mass of Pif80-*x*/aragonite by measuring the nitrogen adsorption isotherms were unsuccessful. On the basis of the SEM images, however, we believe that Pif80-11/aragonite and Pif80-22/aragonite did not have any significant difference in surface area.

### Amount of Pif80-x Bound to Calcium Carbonate

**Figure 3:**
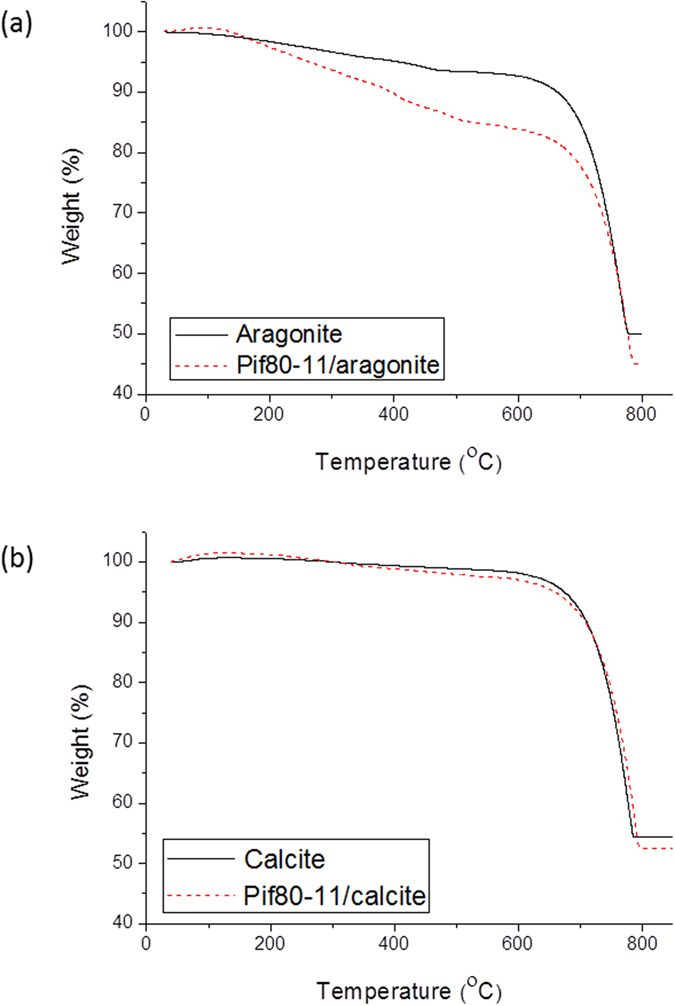
TGA curves of (**a**) Pif80-11/aragonite and (**b**) Pif80-11/calcite. For comparison, the results of calcite and aragonite were also provided.

The precipitates of CaCO_3_ in the gas diffusion experiments were collected by repeatedly conducting centrifugation and washing with deionized (DI) water. Thermo-gravimetric analysis (TGA) was performed to examine the amount of Pif80-*x* bound to the crystallites. As shown in Fig. 3, the TGA curve of Pif80-11/calcite was nearly identical to that of pure calcite. The weight loss in the range of 600 to 800 °C is mainly due to the decomposition of CaCO_3_ to CaO. Similar TGA results were obtained for Pif80-7/calcite and Pif80-22/calcite (Supporting Information, Fig. S6). Hence, we conclude that the interactions between Pif80-*x* and calcite are very limited. On the other hand, the weight loss in the range of 200 to 600 °C, which signifies the combustion of Pif80-*x* peptides, was considerably larger for the Pif80-*x*/aragonite samples (Table 1). Accordingly, the molar amount of peptides adsorbed on the surface and/or trapped between the crystallites is in the order of Pif80-11 > Pif80-22 > Pif80-7 for the Pif80-*x*/aragonite samples. Furthermore, the results of Pif80-*x*/calcite showed that the effect of water adsorption by the peptides, if any, would not offset the trend of the TGA data.

### Nucleation of Aragonite Induced by Pif80-x on β-Chitin Substrate

**Figure 4:**
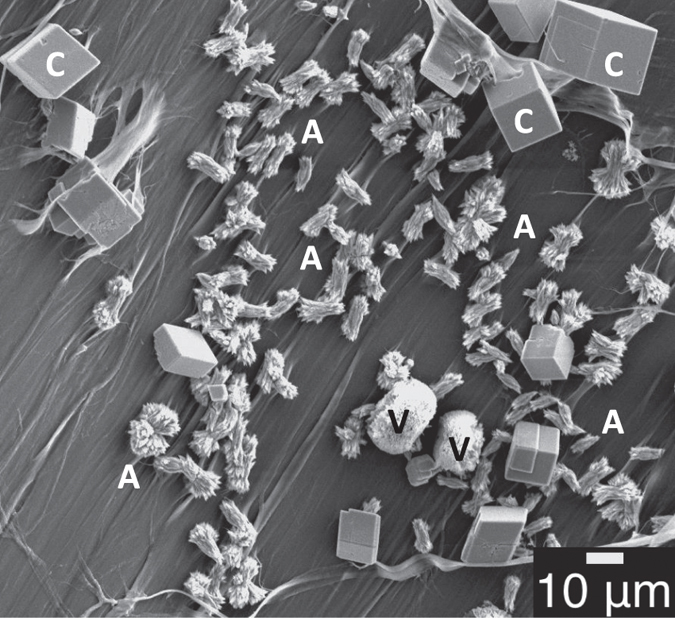
Typical SEM image obtained for the Pif80-11 mineralization assay, using β-chitin as the substrate. The labels C and V denote the crystallites of calcite and vaterite, respectively. The sheaf-like particles next to the labels A are the crystallites of aragonite.

**Figure 5:**
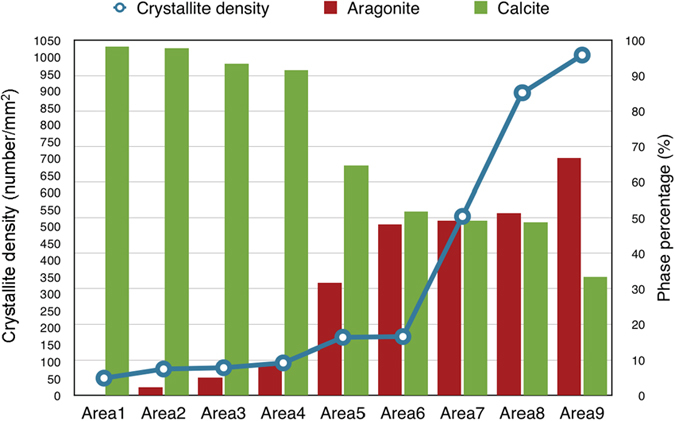
Distribution of the crystallite densities and the relative abundance of aragonite and calcite in nine different regions of the β-chitin substrate of the Pif80-11 mineralization assay. The areas 1 to 9 were designated based on the ascending order of the crystallite densities.

In the foregoing section, we have shown that Pif80-*x* peptides alone cannot induce the nucleation of aragonite. As inspired by the elegant study of Estroff and co-workers^17^, we attempted to verify whether Pif80-*x* could induce the nucleation of aragonite on a substrate of β-chitin. Experimentally, the β-chitin was extracted from squid pens and purified following the reported procedure^27^,^28^,^29^. The gas-diffusion approach described above was adopted without adding any Mg^2+^ ions. Figure 4 shows a typical SEM image obtained for Pif80-11, where the crystallites of both calcite and aragonite were found on the β-chitin surface. In addition, the crystallites of vaterite were also scarcely observed. Following the procedure established in the literature^17^, we carried out a comprehensive analysis of the relative populations of calcite and aragonite in nine different substrate regions (Fig. 5). A clear data trend was observed that a higher density of crystallites was accompanied by a larger population percentage of aragonite. In particular, the populations of aragonite were under 10% in the areas with crystallite densities lower than 100 mm^−2^. When the crystallite densities were higher than 150 mm^−2^, however, the population of aragonite crystallites increased considerably. In the region with the highest crystallite density (1000 mm^−2^), aragonite became the dominant phase (>65%). In a control assay of Pif80-11, where the β-chitin substrate was replaced by a glass slide, only calcite crystallites were found and the crystallite density was approximately 105 ± 20 mm^−2^. The induction of aragonite crystallization was less prominent for Pif80-7 and Pif80-22 (Supporting Information, Fig. S7)

### ^13^C{^1^H} CPMAS spectra of Pif80-22

**Figure 6:**
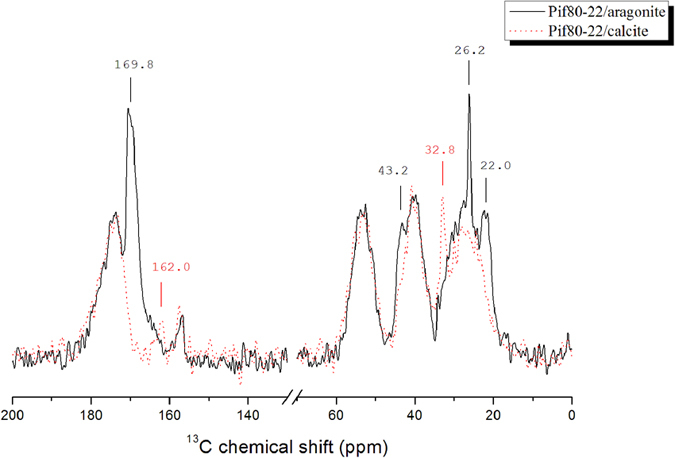
^13^C{^1^H} CPMAS spectra of Pif80-22/aragonite (black line) and the Pif80-22/calcite (red dotted line). The Pif80-22/calcite sample was prepared by mixing Pif80-22 and calcite, followed by lyophilization.

Figure 6 shows the ^13^C{^1^H} CPMAS spectra of Pif80-22/aragonite and the Pif80-22/calcite, both in natural abundance. The Pif80-22/calcite sample was prepared by mixing the Pif80-22 peptides and pure calcite, followed by lyophilization. The peptides of the Pif80-22/calcite sample were presumably in the random coil conformation because the TGA data indicated that Pif80-22 has little interaction with calcite. The signals in the regions of CO and C^α^ are almost identical for Pif80-22/aragonite and Pif80-22/calcite, which implies that the backbone conformation of Pif80-22 adsorbed on aragonite was also random-coiled. The signals at 169.8, 43.2, 26.2, and 22.0 ppm of the Pif80-22/aragonite spectrum were not found in the Pif80-22/calcite spectrum. On the basis of the signal amplitude and the chemical shift, the peak at 169.8 ppm was readily assigned to the carbonate signal of aragonite^30^. The other peaks were tentatively assigned based on the distribution of the chemical shifts of the side-chain carbons of Asp, Arg, and Lys as well as their random coil chemical shift values (Supporting Information, Fig. S8)^31^,^32^. As shown in Table 2, although most of the resonance peaks cannot be assigned unambiguously, it is beyond doubt that the peaks at 26.2 and 22.0 ppm were due to the basic residues of Pif80-22. The signal at 26.2 ppm, with a line width at half maximum equal to 0.72 ppm, was particularly sharp and enhanced in intensity, indicating that basic residues have uniform structures. In addition, the signals at 162.0 and 32.8 ppm were only found in the spectrum of the Pif80-22/calcite and they were again assigned to the basic residues.

### ^13^C−^13^C Correlation spectra of Pif80-11

**Figure 7:**
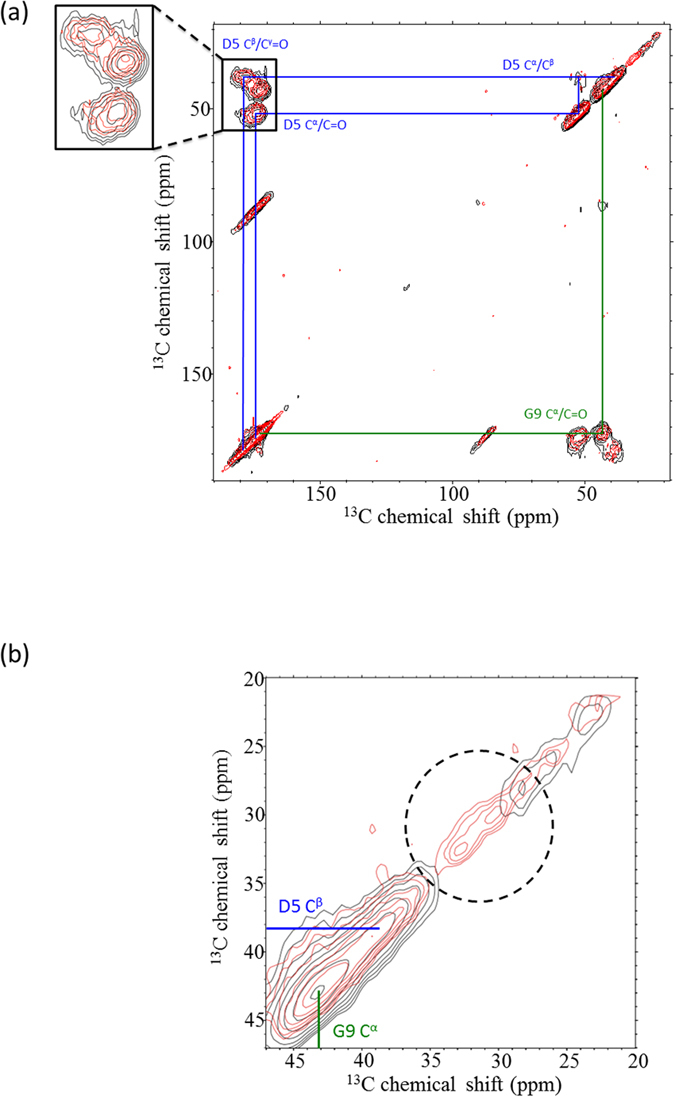
(**a**) Overlay ^13^C−^13^C correlation spectrum of Pif80-11/aragonite (red) and Pif80-11/calcite (black). The residues Asp5 and Gly9 were ^13^C enriched. Number of scans were set to 1024 and 128 for the spectra of Pif80-11/aragonite and Pif80-11/calcite, respectively. The cross peaks at ~85 ppm are due to spinning sidebands. (**b**) Enlarged spectrum in the chemical shift range of 20 to 47 ppm. The dashed circle highlights the signals tentatively assigned to the basic residues Arg and/or Lys.

To shed more light on the backbone conformation of Pif80-*x* upon its binding to aragonite, the ^13^C−^13^C correlation spectrum of Pif80-11/aragonite was acquired, for which the peptides were uniformly ^13^C labeled at the residues Asp5 and Gly9. A control sample, i.e. Pif80-11/calcite was prepared similarly to what has been described for Pif80-22/calcite. Figure 7a shows the overlay spectrum of the Pif80-11/aragonite and Pif80-11/calcite samples. From the cross-peak region highlighted in the inset, the ^13^C chemical shifts (D5: CO, C^α^, C^β^, C^γ^; G9: CO, C^α^) of Pif80-11/aragonite and Pif80-11/calcite were similar to the random coil values (Table S1)^32^. The corresponding line widths at half maximum were larger than 3.5 ppm. Therefore, we conclude that the backbone of the Pif80-11 peptides adsorbed on the surface of aragonite had relatively diverse structures^33^, consistent with our results obtained for Pif80-22/aragonite. As shown in Fig. 7b, in the aliphatic region of the spectrum of Pif80-11/aragonite there were two relatively intense signals at 30.7 and 32.7 ppm, which were not found for Pif80-11/calcite. These natural abundance signals were assigned to the C^β^ of Arg and/or Lys based on their chemical shifts (Supporting Information, Fig. S9). The corresponding line widths at half maximum were equal to 2.6 and 2.1 ppm, respectively (Supporting Information, Fig. S10).

## Discussion

Many proteins associated with biomineralization are intrinsically disordered^34^ and their high degree of structural flexibility might allow the proteins to alter their conformations to recognize a specific polymorph over another^35^. In this work, the CD analysis confirmed that our Pif80-*x* peptides adopted the random-coil conformation in aqueous solutions. Note that the second half of the Pif80-22 sequence is a duplicate of the first half. To investigate whether the sequence of the peptide would modulate its binding affinity to aragonite, we truncated the peptide Pif80-22 in the middle to obtain Pif80-11. After further truncation, the Pif80-7 sequence contains a single copy of the DDRK motif only. That is, each peptide molecule of Pif80-22, Pif80-11, and Pif80-7 contains four, two, and one DDRK units, respectively. As illustrated by the TGA data (Table 1), the total amount of DDRK units loaded on aragonite surface are approximately the same for Pif80-22 and Pif80-11, whereas the loading of DDRK is substantially lower for Pif80-7. The difference between the sequences of Pif80-7 and Pif80-11 is merely a single repeat of the DDRK motif and hence our TGA data confirmed the importance of the DDRK motif in aragonite binding. Given that the initial peptide concentration of the mother liquor was identical for all the three Pif80-*x* peptides, it is interesting to find that Pif80-22 and Pif80-11 had more or less the same amount of DDRK units bound to aragonite. If the interaction between Pif80-*x* and aragonite were solely determined by the number of DDRK units, the amount of the bound DDRK of Pif80-22 should have been two times as much as Pif80-11. It is plausible that the longer peptide chain of Pif80-22 required a substantial entropy loss to adopt the conformation matching the spatial arrangement of the Ca^2+^ and CO_3_^2−^ ions in aragonite. This point is partially justified by the observation that only Pif80-11 can substantially enhance the nucleation rate of calcium carbonate on β-chitin, with stronger effect on aragonite than calcite.

With reference to the ^13^C{^1^H} CPMAS spectra acquired for Pif80-22/aragonite and the Pif80-22/calcite (Fig. 6), the carbonate signal of aragonite (168.9 ppm) was clearly observed in the Pif80-22/aragonite spectrum, whereas the carbonate signal was missing in the Pif80-22/calcite spectrum. Because both aragonite and calcite do not contain any crystal water, these results provided a strong evidence for the atomic-level proximity between Pif80-22 and aragonite. By contrast, the peptides in Pif80-22/calcite had little interaction with the calcite surface. The sharp resonance at 26.2 ppm was observed only when Pif80-22 was bound to aragonite. The relatively narrow line width reveals that there was considerable structural order associated with the side chains. The enhancement of the intensity of this natural-abundance ^13^C signal most likely arose from the anchoring of the residues Arg and/or Lys on the aragonite surface, where the reduced motional dynamics had enhanced the CP polarization transfer. If the binding between Pif80-22 and aragonites were simply dominated by electrostatic attractions, the residues residing on the crystal surface should have been structurally diverse in nature, which would not have resulted in any narrow signals. On the other hand, it is not trivial to rationalize why the two signals at 162.0 and 32.8 were only observed in the spectrum of Pif80-22/calcite but not in that of Pif80-22/aragonite. Nonetheless, this observation is largely in line with the expectation that the side-chain conformation of Pif80-22 would change upon its binding to aragonite surface.

For the NMR data of Pif80-11/aragonite, the natural abundance signals at 30.7 and 32.7 ppm were assigned to the basic residues. Although Pif80-11 and Pif80-22 have exactly the same chemical composition, the side-chain chemical shifts of Pif80-11/aragonite and Pif80-22/aragonite are rather different. In other words, the basic residues of Pif80-11/aragonite and Pif80-22/aragonite have different side-chain conformations. Altogether, we conclude that the side chains of the positively charged residues of Pif80-22 and Pif80-11 had considerable structural order induced upon its binding to the surface of aragonite, even though their backbone conformation remained to be disordered. A similar structural ordering of the side chains has been reported for three nacre-associated polypeptides, viz., AP24N, AP7N, and N16N, upon their affiliation with calcite^16^. For the basic polypeptide N16N (AYHKKCGRYSYCWIPYDIERDRYDNGDKKC, pI = 8.9), a control sample N16NN was prepared in which the acidic residues Glu and Asp were replaced by Asn and Gln, respectively. A comparison of the carbon K-edge XANES spectra of N16N and N16NN strongly suggests that the spectral difference is due to ordering of the side chains of the acidic residues^16^, not the basic residues. For the sake of spectral resolution, we only isotopically enriched the residue Asp5 for our ^13^C NMR measurements. Strictly speaking, we therefore cannot rule out the possibility that the acidic residues Asp1 and Asp2 also possess some structural ordering in the sample of Pif80-*x*/aragonite. In fact, the ^43^Ca NMR data acquired for osteocalcin-apatite have revealed that the structural environment of calcium ions on the mineral surface will be strongly perturbed by the negatively charged residues of osteocalcin such as aspartic acid and/or γ-carboxyglutamic acid^36^. Nonetheless, the different binding behavior among Pif80-*x*/calcite and Pif80-*x*/aragonite reflects that the interfacial interactions depend both on the peptide sequence and on the surface structure of the mineral substrate.

Most proteins associated with the biomineralization of CaCO_3_ are acidic and they are rich in Asp and Glu, which are negatively charged under physiological conditions^37^. However, some concerns have been raised against the common belief that Asp-rich proteins play a central role in the shell matrix of nacre^10^. As a matter of fact, a basic protein has recently been found in the nacreous layer of oyster shells^38^. Positively charged polymers can significantly modulate the crystal morphology of vaterite^39^ and calcite^40^. An engineered heptapeptide containing positively charged residues had demonstrated preferential binding affinity to vaterite over aragonite^41^. Computational studies also revealed that the arginine in ovocladin-17 may be the most important anchors of the protein to the surface of calcite^42^,^43^. In the present work, we indeed found that the basic residues of Pif80-22 and Pif80-11 are important in binding the peptides to the aragonite surface. Together with the fact that the Lys and Arg residues of salivary protein statherin bound to hydroxyapatite are in close proximity to the crystal surface^44^,^45^, we believe the role of basic residues as anchors in protein-mineral interaction may be a more general phenomenon than previously thought.

## Conclusions

Three fragmental peptides derived from Pif80 protein, viz. Pif80-7, Pif80-11, and Pif80-22, were synthesized and they were intrinsically disordered in aqueous solutions. Pif80-*x* peptides alone could not instigate the nucleation of aragonite, and they would bind to aragonite but not to calcite. In the presence of β-chitin substrate, Pif80-11 could facilitate the nucleation of aragonite. This synergistic effect between Pif80-11 and β-chitin on the nucleation rate of aragonite was not observed in Pif80-7 and Pif80-22. Thus, the effect was sequence dependent and polymorphic selective. Solid-state NMR results showed that the backbone conformation of the peptides bound to aragonite was random-coiled but the side chains of Arg and/or Lys were well structured on the surface of the crystallites.

## Methods

### Preparation of Pif80-x Peptides

All chemicals were obtained from Acros unless stated otherwise. The target peptides with C-terminal amidation were synthesized by standard Fmoc method^46^. The scale of synthesis was 0.1 mmol. Pif80-7 and Pif80-11 were synthesized manually, whereas Pif80-22 was synthesized by microwave-assisted synthesizer (Liberty Blue, CEM Corporation) with Fmoc-PAL-PEG-PS resin (Life Technology), 10% (vol/vol) piperazine with 0.1 M HOBt in 1:9 ethanol/NMP solution as deprotection agent, N,N′-Diisopropylcarbodiimide in DMF as activator, and ethyl 2-cyano-2-(hydroxyimino) acetate (Merck) in DMF as activator base. Single coupling was performed for all amino acids at 90 °C for 4 minutes. The deprotection steps were conducted for 5 minutes, two times at room temperature without microwave irradiation. After drying the resin with dichloromethane, the crude peptides were cleaved from the resin with a reagent mixture of 92.5% trifluoroacetic acid, 2.5% triisopropylsilane, 2.5% 3,6-dioxa-1,8-octanedithiol (Sigma Aldrich), and 2.5% DI water for 2.5 hours. The crude peptides were then precipitated in methyl tert-butyl ester precooled at −20 °C. Crude product was dried and then purified by high-performance liquid chromatography at room temperature with a Vydac C18 column, utilizing a water/acetonitrile gradient with 0.1% TFA.

### Calcium Carbonate Crystallization Assay

Crystallization experiments were conducted by a gas-diffusion method. The samples of Pif80-*x*/calcite were prepared as follows. Two glass vials containing 1 g of (NH_4_)_2_CO_3(s)_ and a 5 mL solution of 100 mM CaCl_2_ and selected amount of Pif80-*x* were placed in a two-liter desiccator for 24 hours, where the pressure was initially reduced to 120 Torr. All sample vials were covered by Parafilm with pinholes and the solution was under continuous stirring. Precipitates were collected by repeat centrifugation and washing with DI water three times, followed by lyophilization. To prepare the samples of Pif80-*x*/aragonite, the same procedure was repeated with the solution of 100 mM of CaCl_2_, 100 mM of MgCl_2_, and 0.47 mM of Pif80-*x*. On the other hand, one sample batch of Pif80-11/aragonite was prepared using the peptides with uniform ^13^C labeling at the residues Asp5 and Gly9. For comparison, a control sample was prepared by mixing 8 mg of the labeled Pif80-11 peptides and 72 mg of calcite in DI water, followed by lyophilization.

### Mineralization Assay on the β-Chitin Substrate

Beta-chitin was extracted from squid pens. Fresh pens were washed thoroughly with DI water and cut into small pieces (~0.5 cm^2^/piece). Each batch of crude pens (0.1 g) was refluxed with 15 mL of 1 M NaOH solution at 65 °C for 24 hours. This procedure was repeated once with fresh NaOH solution. The purified β-chitin pieces were rehydrated by 5 mM CaCl_2(aq)_ for two hours prior to gas diffusion. Each piece of the β-chitin was immersed in 2 mL of 5 mM CaCl_2(aq)_ and 5 μM Pif80-*x* peptides in a glass Petri dish which was covered by Parafilm with pinholes. The gas diffusion experiments were carried out without the initial pressure reduction. The mineralized samples were rinsed by DI water and then lyophilized.

### Characterization

The spectra of CD were collected on a Jasco J815 spectrometer with a cell of 1 mm path length. The peptide concentration was 0.1 mM and the pH was adjusted to 8.5. XRD analysis was performed on a Philips X’pert diffractometer using Cu K*α* radiation (*λ* = 1.5418 ang). TGA measurements were conducted on a Mettler Toledo TGA star system or on a NETZSCH TG209 thermal gravimetric analyzer. The temperature was steadily increased from room temperature to 900 °C using a heat rate of 10 °C/min. SEM images were taken on a JEOL JSM-7600F field emission scanning electron microscope. The samples of CaCO_3(*s*)_ were coated with platinum by low-vacuum sputtering at 10 mA for 60 seconds.

### Solid-State NMR

All NMR experiments were carried out at ^13^C and ^1^H frequencies of 150.92 and 600.21 MHz, respectively, on a wide-bore Bruker Avance III spectrometer equipped with a 4-mm double-resonance magic-angle spinning probe. The rotor packed with each sample of ~90 mg was spun at 13 kHz. ^13^C chemical shifts were externally referenced to tetramethylsilane (TMS) using adamantane as the secondary reference. The initial ^13^C magnetization was prepared by ^13^C{^1^H} cross polarization (CP) with a contact time of 1 ms, during which the rf field of the ^1^H channel was linearly ramped from 35.0 to 50.0 kHz and that of ^13^C was optimized at 30.3 kHz. For the ^13^C−^13^C correlation experiment, the ^13^C rf field and mixing time for the radio frequency driven recoupling (RFDR)^47^ was set to 75 kHz and 2.46 ms, respectively. The States-TPPI scheme was implemented for the quadrature detection in the indirect dimension. A total of 64 increments were acquired at steps of 38.5 μs. Spectral analysis of the ^13^C{^1^H} CPMAS spectra were carried out by DMFit^48^.

## Additional Information

**How to cite this article**: Du, Y.-P. *et al*. Study of Binding Interaction between Pif80 Protein Fragment and Aragonite. *Sci. Rep.* **6**, 30883; doi: 10.1038/srep30883 (2016).

## Supplementary Material

Supplementary Information

## Figures and Tables

**Table 1:** Summary of the TGA data of Pif80-*x*/aragonite.

Pif80-*x* (molar mass)	weight loss^a^[%]	peptide adsorbed per unit mass of CaCO_3_^b^ × 10^−5^ mole/g	DDRK adsorbed per unit mass of CaCO_3_^c^ × 10^−5^ mole/g
Pif80-7 (773.84)	2	3	3
Pif80-11 (1288.38)	7 ± 1	6 ± 1	12 ± 2
Pif80-22 (2559.72)	9 ± 2	4 ± 1	16 ± 4

^a^Weight loss in the range of 200 to 600 °C. Error estimation was carried out by repeat measurements.

^b^Mass ratio of peptide and CaCO_3_ divided by the molar mass of peptide. Contribution from the structural water was ignored.

^c^Each peptide molecule of Pif80-22, Pif80-11, and Pif80-7 contains four, two, and one DDRK units, respectively.

**Table 2:** Assignment of the ^13^C signals uniquely found in Pif80-22/aragonite, Pif80-22/calcite, and Pif80-11/aragonite.

		Pif80-22/aragonite				Pif80-22/calcite		Pif80-11/aragonite	
Resonances (ppm)		169.8	43.2	26.2	22.0	162.0	32.8	32.7	30.7
aragonite		**							
Gly	C^α^ (43.66 ± 1.27)^a^, 43.4^b^		*						
Asp	C^α^ (53.00 ± 2.04), 52.5								
	C^β^ (39.17 ± 1.61), 39.4		+						
Arg	C^β^ (28.96 ± 1.82), 29.2			+			+	+	*
	C^γ^ (25.51 ± 1.18), 25.4			*					
	C^δ^ (41.46 ± 0.89), 41.6		+						
	C^ζ^ (158.33 ± 2.98), 157.8					*			
Lys	C^β^ (31.08 ± 1.77), 31.4			+			*	*	*
	C^γ^ (23.20 ± 1.12), 23.0				**				
	C^δ^ (27.25 ± 1.08), 27.3			*					+
	C^ε^ (40.19 ± 0.83), 40.2								

^a^Distribution of chemical shifts of the side-chain carbons relevant to our spectral assignment^31^, where the margin of error represents one standard deviation.

^b^Random coil chemical shifts of the corresponding side-chain carbons^32^.
