# Supplementary material for: Study of Binding Interaction between Pif80 Protein Fragment and Aragonite
Source: Sci Rep. 2016 Aug 3;6:30883. doi: 10.1038/srep30883 (PMC4971512; doi:10.1038/srep30883)
Supplement: Supplementary Information [file srep30883-s1.pdf]

Supporting Information

Title: Study of Binding Interaction between Pif80 Protein Fragment and Aragonite

Yuan-Peng Du,[a] *Hsun-Hui Chang,*[a] *Sheng-Yu Yang,*[a] *Shing-Jong Huang,*[b] *Yu-Ju Tsai,*[c] *Joseph Jen-Tse Huang,*[c] *Jerry Chun Chung Chan*[a]*

[a] Department of Chemistry, [b] Instrumentation Center, National Taiwan University, No. 1, Section 4, Roosevelt Road, Taipei 106, Taiwan; [c] Institute of Chemistry, Academia Sinica, No. 128, Sec. 2, Academia Road, Nankang, Taipei 115, Taiwan

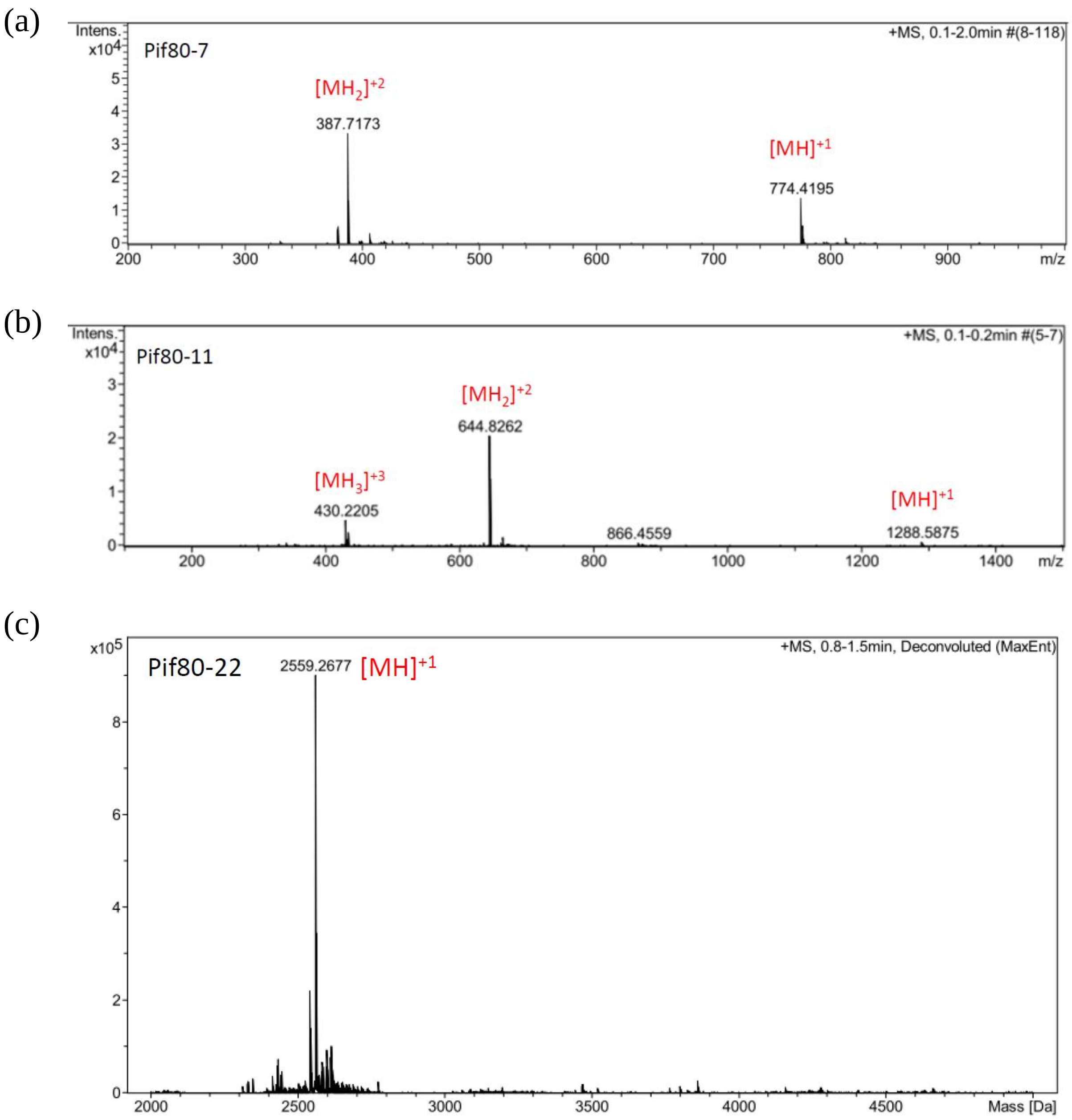

Figure S1. Mass spectra of (a) Pif80-7, (b) Pif80-11, and (c) Pif80-22 collected by ESI-MS. The expected molecular masses were 773.403, 1287.653, and 2558.291 Da, respectively.

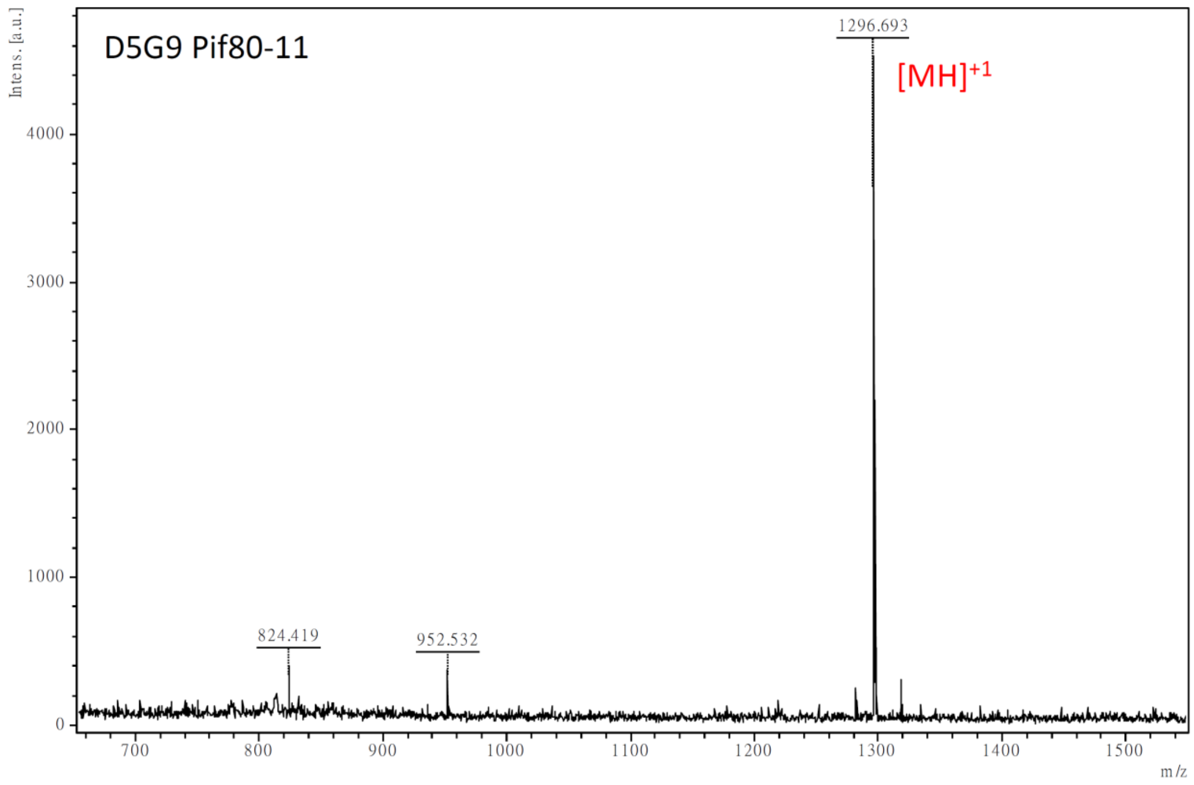

Figure S2. MALDI-TOF spectrum of Pif80-11 which was ^{13}C and ^{15}N enriched at the residues of Asp5 and Gly9. The expected molecular mass was 1295.667 Da.

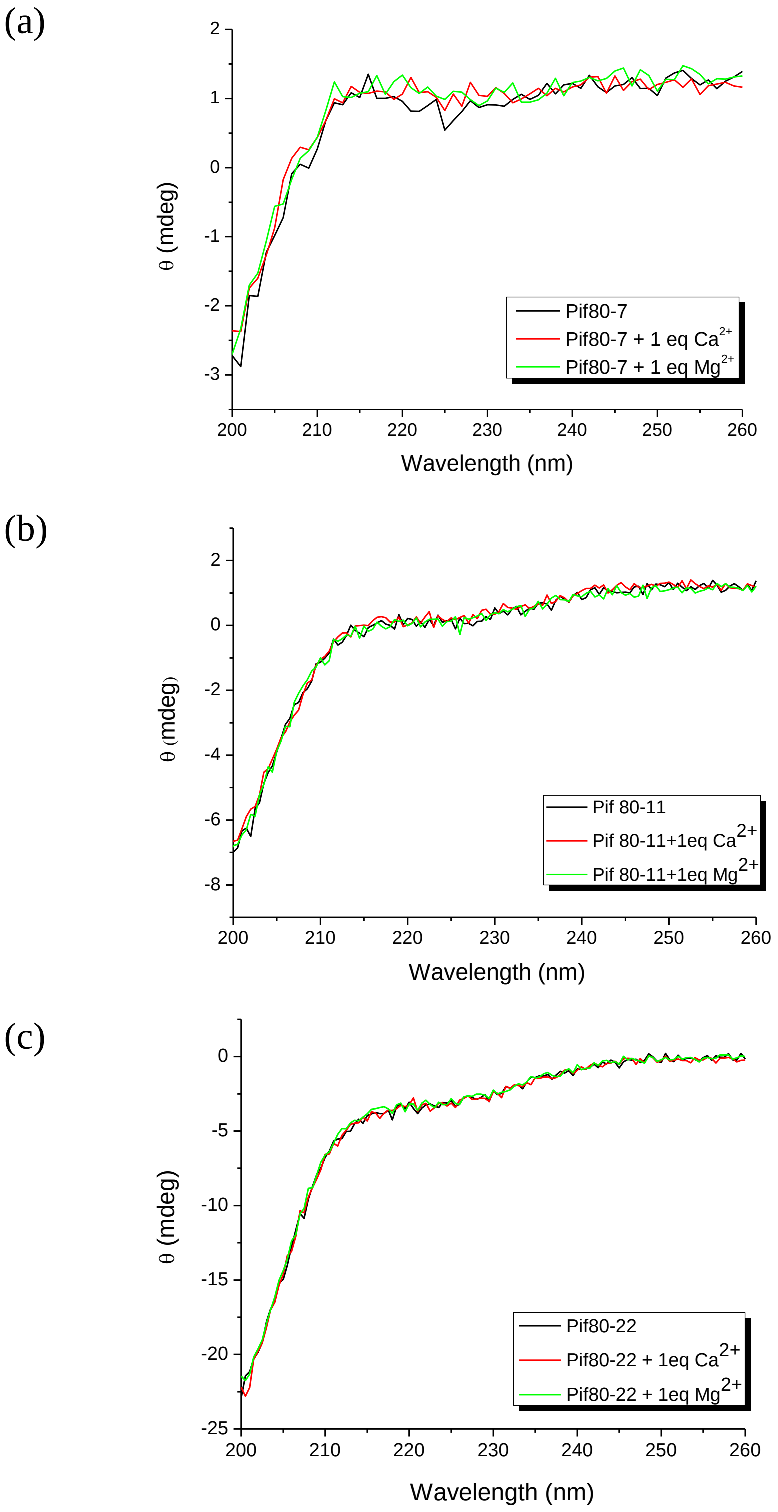

Figure S3. CD spectra of (a) Pif80-7, (b) Pif80-11, and (c) Pif80-22 in Tris-buffer solutions.

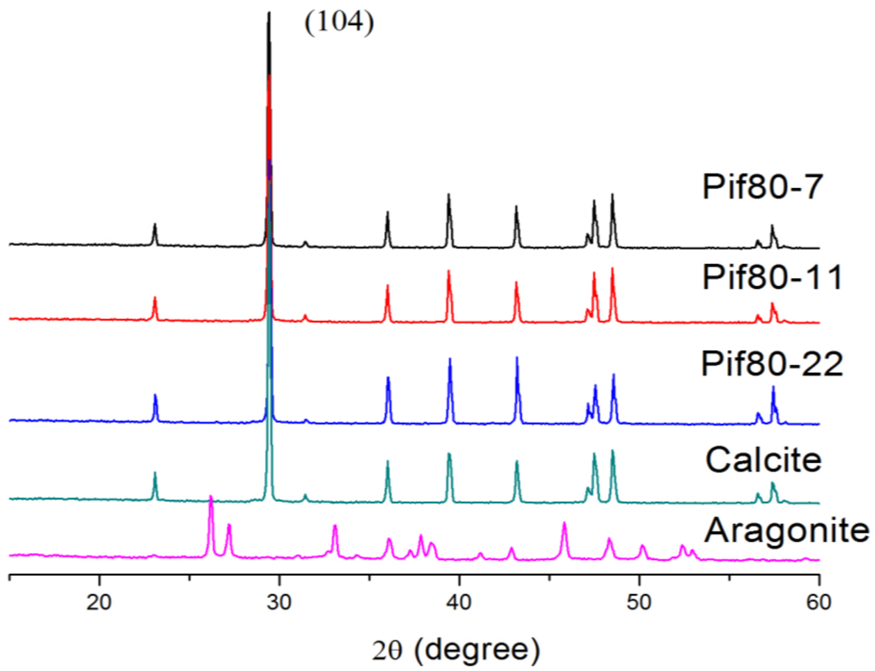

Figure S4. XRD patterns acquired for Pif80-*x*/calcite. The results of pure calcite and aragonite were included for comparison. Before the XRD measurements, all samples were washed with deionized water and then lyophilized. The (104) diffraction peaks were analyzed with the Scherrer formula and the crystal grain sizes of Pif80-7/calcite, Pif80-11/calcite, and Pif80-22/calcite were estimated to be 55, 57, and 69 nm, respectively.

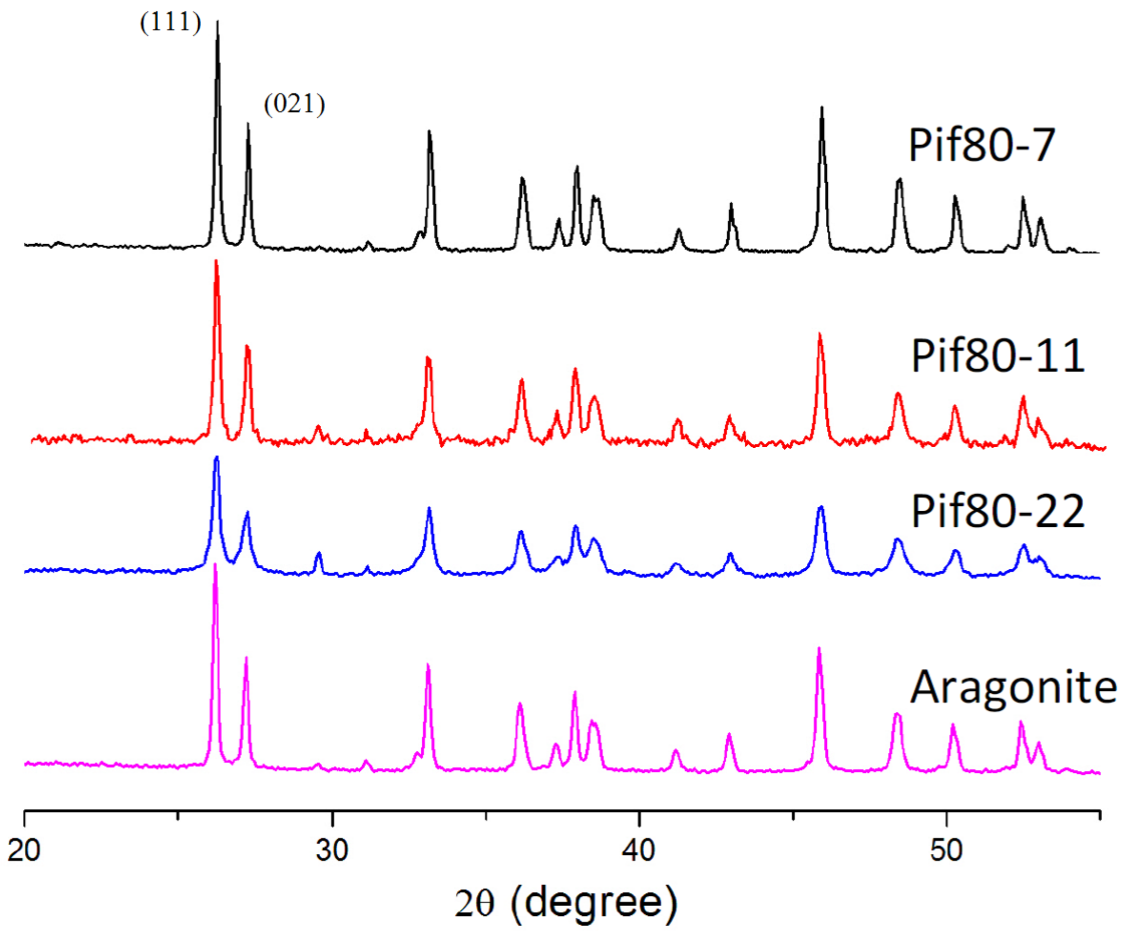

Figure S5. XRD patterns acquired for Pif80-*x*/aragonite. The pattern of pure aragonite was included for comparison. The (111) and (021) diffraction peaks were analyzed with the Scherrer formula and the average crystal grain sizes of Pif80-7/aragonite, Pif80-11/aragonite, and Pif80-22/aragonite were estimated to be 57, 45, and 35 nm, respectively.

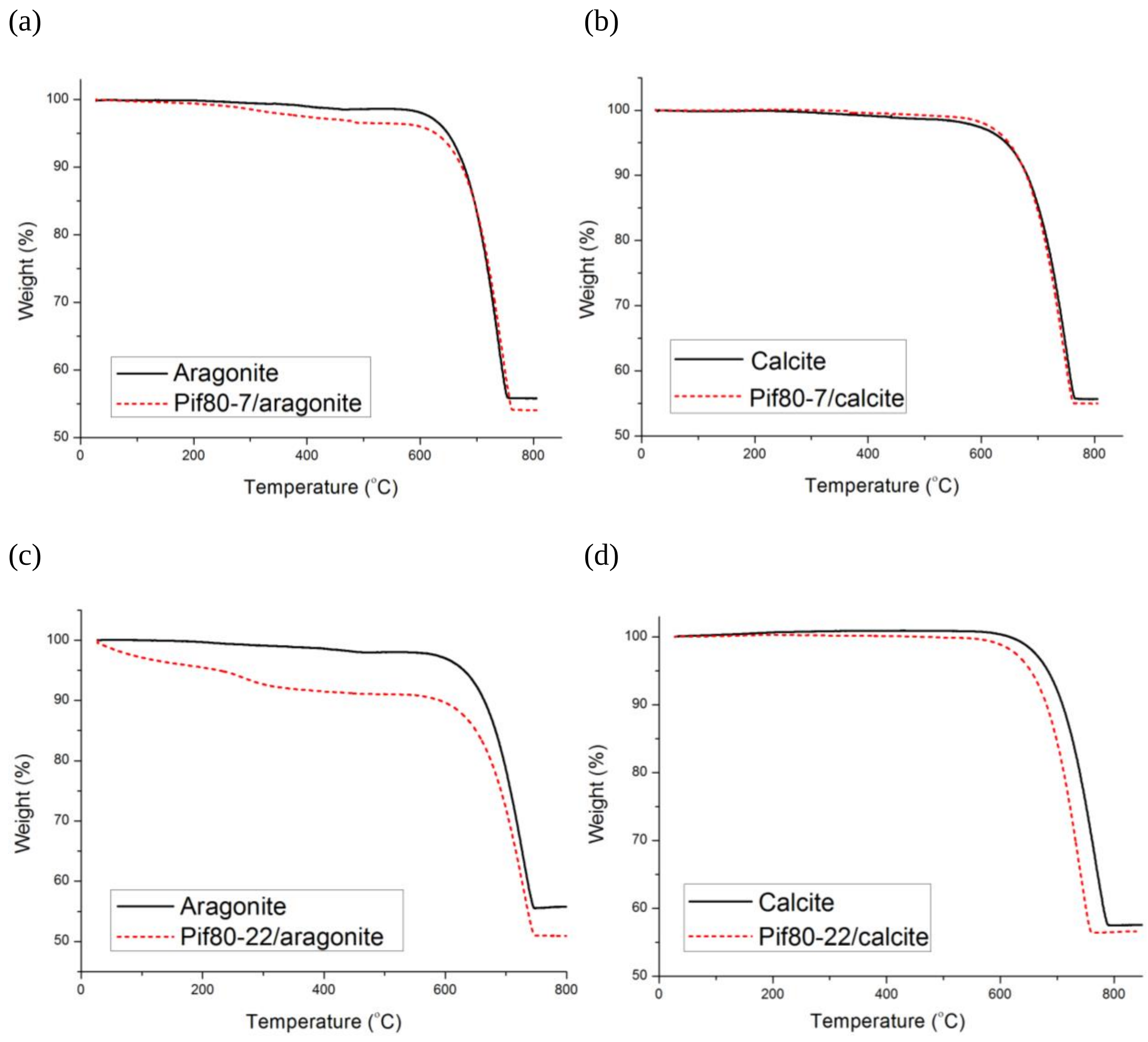

Figure S6. TGA results of (**a**) Pif80-7/aragonite, (**b**) Pif80-7/calcite, (**c**) Pif80-22/aragonite, and (**d**) Pif80-22/calcite.

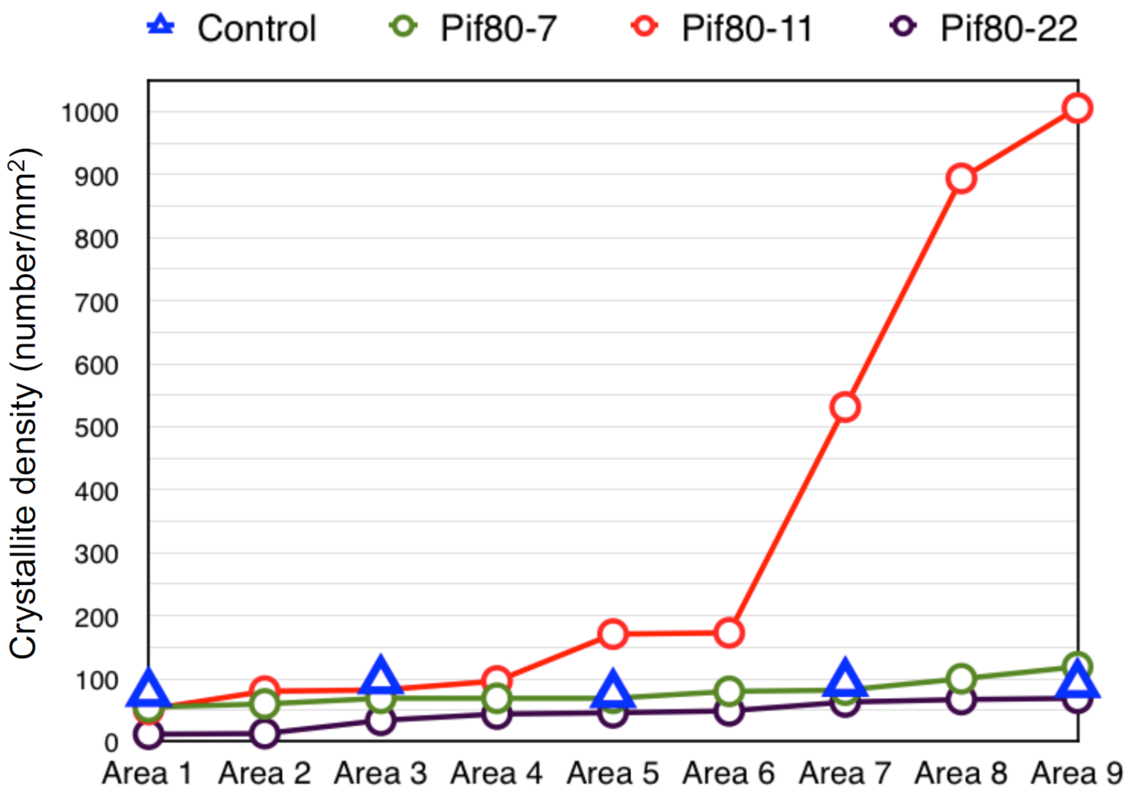

Figure S7. Comparison of the crystallite densities obtained for the Pif80-*x* mineralization assay. For the control assay of Pif80-11, the β-chitin substrate was replaced by a glass slide.

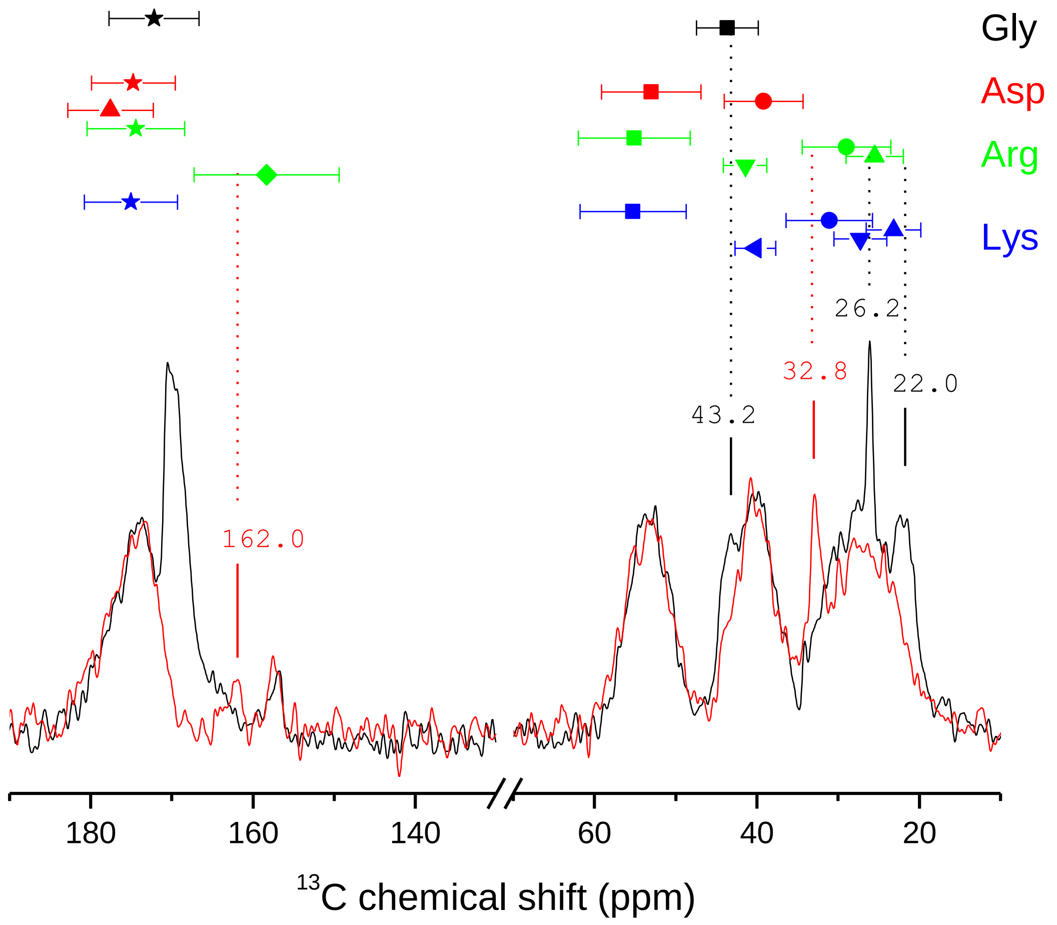

Figure S8. $^{13}C\{^1H\}$ CPMAS spectra of Pif80-22/aragonite (black line) and the Pif80-22/calcite (red line). The symbols of stars, squares, circles, up triangles, down triangles, diamonds, and left triangles indicate the averaged ^{13}C chemical shift of CO, C^{α}, C^{β}, C^{γ}, C^{δ}, C^{ζ}, and C^{ε} of the relevant amino acids. The bars denote the distribution of three standard deviations. The chemical shift data were obtained from the filtered set of the Biological Magnetic Resonance Data Bank (http://www.bmrb.wisc.edu/), where entries containing aromatic and/or paramagnetic ligands were discarded.

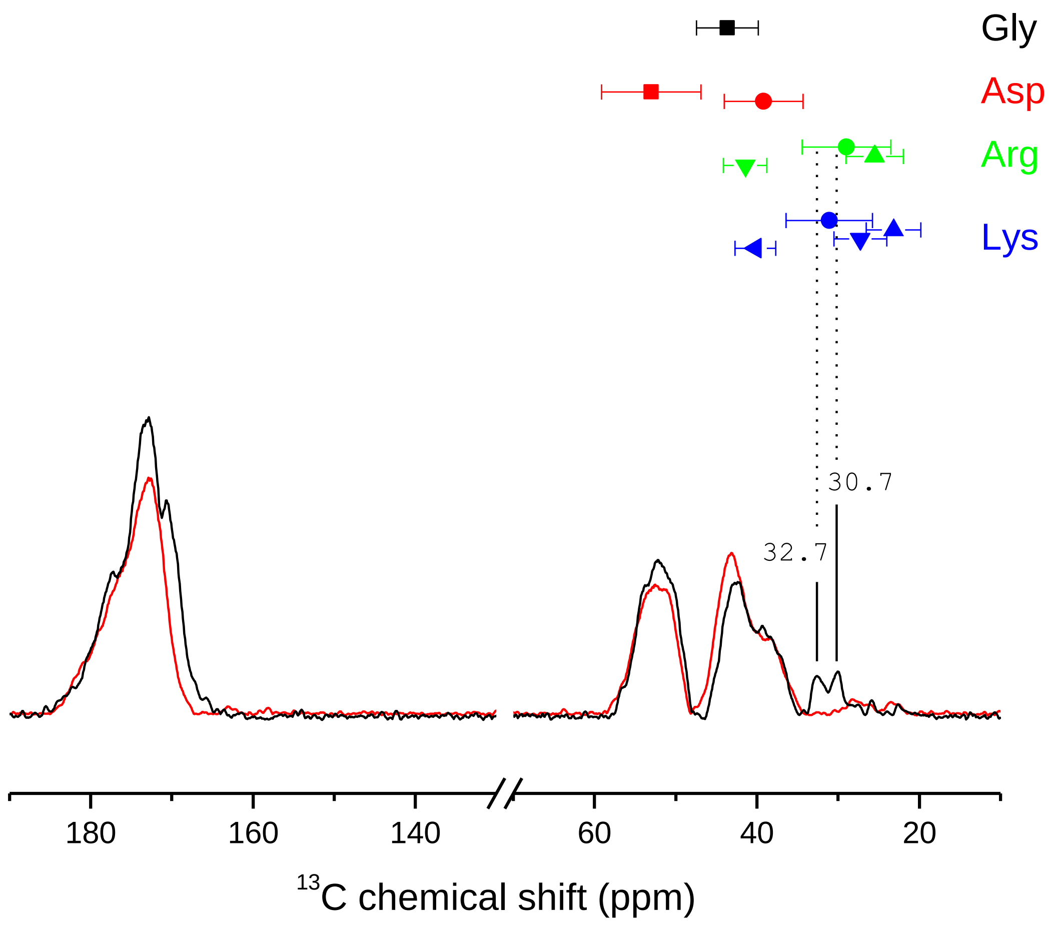

Figure S9. Projections of the ^{13}C homonuclear correlation spectra of Pif80-11/aragonite (black line) and Pif80-11/calcite (red line). Other details are referred to the caption of Figure S8.

(a)

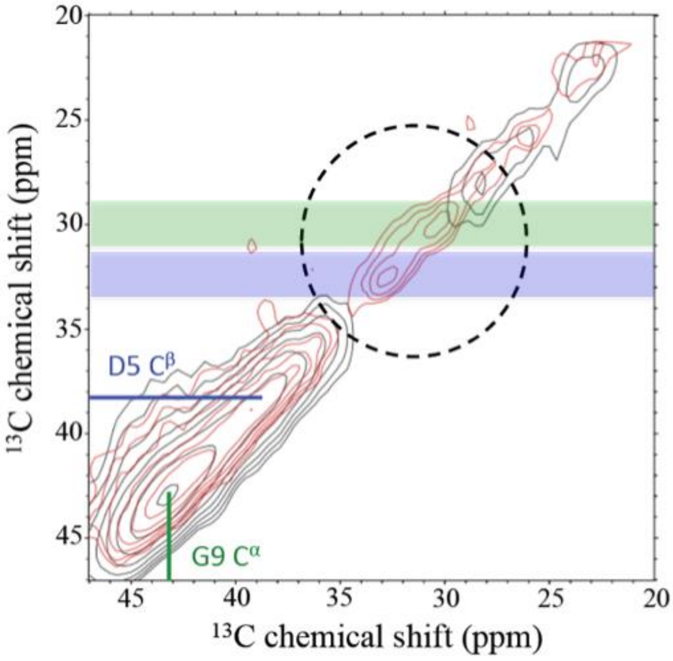

(b)

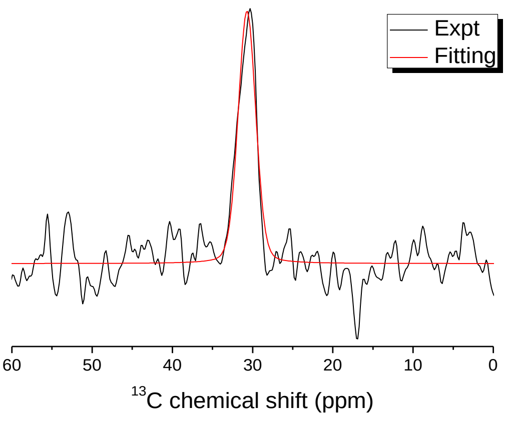

(c)

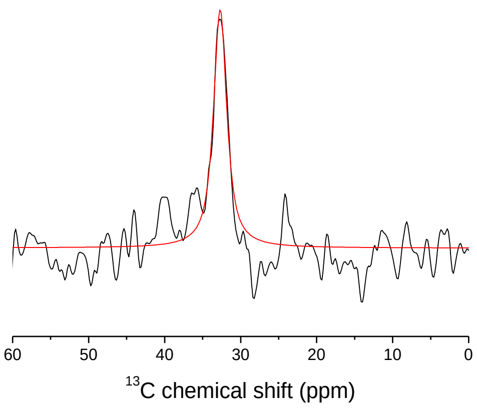

Figure S10. Spectral deconvolution of the ^{13}C signals observed in Pif80-11/aragonite but not in Pif80-11/calcite. (**a**) Sum projections of the 2D spectrum calculated separately in the green and blue regions. Spectral fittings of the projections of the (**b**) green region and (**c**) blue regions. The corresponding line widths at half maximum of the signals at 30.7 and 32.7 ppm were equal to 2.6 and 2.1 ppm, respectively.

Table S1. Chemical shifts of Pif80-11/aragonite and Pif80-11/calcite with ^{13}C enrichment at the residues of Asp5 and Gly9. All data were referenced to TMS in ppm.

	Pif80-11/aragonite		Pif80-11/calcite (Control)		Random coil*	
	D5	G9	D5	G9	D	G
CO	173.9	172.0	173.6	172.4	174.6	173.2
C^{α}	51.9	42.2	51.3	43.3	52.5	43.4
C^{β}	38.8		38.6		39.4	
C^{γ}	178.0		178.3		178.3	

* The chemical shifts of random coils were taken from the literature (D. S. Wishart, C. G. Bigam, A. Holm, R. S. Hodges, B. D. Sykes, Journal of Biomolecular NMR 1995, 5, 67–81).
